# On some fundamental challenges in monitoring epidemics

**DOI:** 10.1098/rsta.2021.0117

**Published:** 2022-01-10

**Authors:** Vaiva Vasiliauskaite, Nino Antulov-Fantulin, Dirk Helbing

**Affiliations:** ^1^ Computational Social Science, ETH Zürich, Zürich, Switzerland; ^2^ Complexity Science Hub Vienna, Wien, Austria

**Keywords:** epidemic modelling, complex systems, network theory, computer simulation, data science, statistics

## Abstract

Epidemic models often reflect characteristic features of infectious spreading processes by coupled nonlinear differential equations considering different states of health (such as susceptible, infectious or recovered). This compartmental modelling approach, however, delivers an incomplete picture of the dynamics of epidemics, as it neglects stochastic and network effects, and the role of the measurement process, on which the estimation of epidemiological parameters and incidence values relies. In order to study the related issues, we combine established epidemiological spreading models with a measurement model of the testing process, considering the problems of false positives and false negatives as well as biased sampling. Studying a model-generated ground truth in conjunction with simulated observation processes (virtual measurements) allows one to gain insights into the fundamental limitations of purely data-driven methods when assessing the epidemic situation. We conclude that epidemic monitoring, simulation, and forecasting are wicked problems, as applying a conventional data-driven approach to a complex system with nonlinear dynamics, network effects and uncertainty can be misleading. Nevertheless, some of the errors can be corrected for, using scientific knowledge of the spreading dynamics and the measurement process. We conclude that such corrections should generally be part of epidemic monitoring, modelling and forecasting efforts.

This article is part of the theme issue ‘Data science approaches to infectious disease surveillance’.

## 1. Introduction

For thousands of years, the epidemic spreading of diseases has been one of the greatest threats to societies around the world. Now, with increased mobility, diseases tend to spread faster and wider due to air traffic and, in fact, often globally. The resulting pandemics can cost the lives of millions of people and disrupt social, economic, public, political and cultural life.

These challenges are increasingly countered with new measurement and vaccination technologies [1]. In particular, digital technologies have enabled data-driven and AI-based methods [2–4], which have become quite popular. Some organizations even envision a future with ubiquitous health measurements, using, for example, in-body sensors [5].

Data analytics is also used for non-pharmaceutical interventions to handle an epidemic, such as lockdowns, social distancing, or the use of face masks. The effects of such interventions have been studied extensively to understand the possible impact on the trajectory of epidemics, including the current COVID-19 pandemic [6–12].

Already now, one can say that a data-driven approach has spread widely. This does often assume that the overall picture of the situation will become more accurate with a greater amount of data. Although one may expect that enough data will reveal the truth by itself, it is known that conventional Big Data analytics may face some issues, which are summarized in §A of the electronic supplementary material. Consequently, an improper use of Big Data may result in learning biased patterns that can imply problems and amplify uncertainties. When applied to policy making regarding epidemics, it may also sometimes lead to unnecessary lockdowns or affect the efficiency of measures taken to counter a disease.

In the following, we will investigate to what extent such issues may undermine an accurate assessment of the state of epidemics, even if a large amount of data is available. This is important, as responding to measurement-based predictions rather than to current data when taking proactive measures is becoming increasingly common. For example, one response to an anticipated increase of infections may be to engage in more testing to capture the expected rise. The motivation for this is clear: if infections are underestimated, hospitals might not be able to handle the number of emergencies, while an overestimation may lead to unnecessary lockdowns with severe socio-economic consequences. In both cases, unnecessary loss of lives may occur. However, is it always better to make more tests? Not necessarily so: as we will discuss, there may be undesirable side effects.

The precondition for accurate predictions is to have reliable measurement methods judging the actual state of epidemics well. Currently used epidemic modelling methods try to describe the disease dynamics with systems of differential equations, meta-population models or individual level simulations [13–18]. Such models are, for example, used to assess the influence of population density, demographic factors, mobility or social interactions on the actual disease dynamics [6–8,18]. Data-driven or machine learning models make fewer assumptions about the actual dynamics and are applicable to a broader range of prediction problems, but they come at the cost of less explainability. Of course, it is also possible to combine classical modelling approaches with machine learning [19].

It seems, however, that in many policy decisions today, issues related to measurement processes are not yet sufficiently considered. Corrections for false positives and false negatives, even though these are a general problem, have been proposed in relatively few publications. For example, the authors of [20] used a statistical testing model with bias and testing errors along with estimates of certain population types in a compartmental model (e.g. the number of recovered unreported cases). Similarly, the authors of [21] used a Bayesian hierarchical model to evaluate the true number of cases from non-representative testing results. In [22], the authors studied implications of having uncertainty or intrinsic testing errors (false positive and false-negative rates). In [23], the authors proposed a method to pool mortality data from European countries to estimate the true incidence rate of the COVID-19 pandemic. [24] used a semi-Bayesian probabilistic bias analysis to account for incomplete testing and imperfect diagnostic accuracy, emphasizing the great under-estimation of COVID-19 severity in the USA. All of these works use real noisy epidemic data without a known ground truth to evaluate the performance of their proposed methods.

By contrast, our contribution will highlight problems related to monitoring epidemics by measurement processes. As it turns out, it is dangerous to assume that data-driven approaches would be largely exact, or that more measurements or tests or data would automatically give a better picture. Therefore, we will demonstrate the possible pitfalls of such an approach in the following—by analysing the estimation errors resulting from measurement errors, mean value approximations, randomness and network interactions. For the sake of quality assessment, we assume an epidemic model as ground truth.

Specifically, we analyse the limits to inferring the hidden number of infectious people within the population by means of established measurement procedures. Our measurement model considers testing errors, and it is assessed for the case when the true state of epidemics is well described by a stochastic epidemiological simulation in a networked population. In this controlled setting, we can quantify a mean-field correction and determine its—often quite significant—deviation from the truth in the presence of parameter uncertainties regarding intrinsic testing errors, testing biases, and network effects. We also discuss how to correct for biases with mean-field and Bayesian approaches, where the latter may incorporate both testing-related and network-related information. In summary, we present an investigation and quantification of fundamental limitations in estimating the state of epidemics.

## 2. Epidemic models

Depending on the characteristics of a disease, there are different compartmental models in epidemiology that aim to reflect different aspects of disease spreading and recovery by coupled differential equations. These are mean value equations implicitly assuming that the infection process is well characterized by averages and correlations do not matter. In the following, we will present the SEIRD model used in our analysis, whereas the simpler SIR and SEIR models are presented in §B of the electronic supplementary material.

### SEIRD model

The SEIRD model [25] assumes that some people recover from the disease and are immune after recovery, while some people die. Besides the conventional categories of Susceptible, Infectious and Recovered, it considers the categories of Died and Exposed (i.e. infected, but not yet infectious, typically without symptoms). Intuitively, the numbers at time t are represented by S(t), I(t), R(t), D(t) and E(t). The increase in the number of Exposed E is proportional to the number of Susceptible S and the number I of those who can infect them. The proportionality constant is b. It depends on how infectious the disease is and the average number of contacts per individual and unit time. Exposed turn to Infectious with a rate a. Infectious recover with a rate c and die with a rate d. The differential equations describing the change of their numbers in the course of time are

2.1 $$\begin{array}{r} \dot{S}=-bSI/N, \end{array}$$

2.2 $$\begin{array}{r} \dot{E}=bSI/N-aE, \end{array}$$

2.3 $$\begin{array}{r} \dot{I}=aE-cI-dI, \end{array}$$

2.4 $$\begin{array}{r} \dot{R}=cI \end{array}$$

2.5 $$\begin{array}{r} \;\text{and}\;\dot{D}=dI. \end{array}$$

Moreover, we have the normalization equation

2.6 S(t)+E(t)+I(t)+R(t)+D(t)=N,

where N is the initial population size. Note that in other publications the parameters *a*, *b*, *c*, *d* are often represented by Greek letters such as α,β,γ,δ.

## 3. Measuring the state of an epidemic

The core question of this paper is: how well can the state of epidemics be measured using common test and monitoring methods? When discussing this, we must consider that all test methods have false positive rates and false negative rates [26], even though they may be small. This also applies to virtual measurement methods such as those based on inference from tracing data. We assume that Infectious I are being tested with probability p and people without symptoms (S, E and R) with probability q. When the testing is focused on infectious people with symptoms (I), we will have q≤p or even q≪p.

In the following, for the sake of illustration, we will focus on the SEIRD model as ground truth. Furthermore, let the false-positive rate (FPR) when healthy people are tested be α, and the false-negative rate (FNR) be β when Infectious are tested, but $\beta^{\prime }$ when Exposed are tested. Under these circumstances, the expected number of positive tests is

3.1 $$N_{p}(t)=(1-\beta )pI(t)+(1-\beta^{\prime })qE(t)+\alpha q[S(t)+R(t)].$$

Accordingly, the assumption that the number $N_{p}(t)$ of positively tested is proportional to the number I(t) of Infectious holds only if $\beta^{\prime }=1$ and α=0 (which is unrealistic [26]), or if q=0 (i.e. if ‘healthy’ people are not being tested). Otherwise, $N_{p}(t)$ can be quite misleading. In fact, the term αq(S+R) may dominate the other terms, if a large number of people without symptoms are being tested (corresponding to ‘mass testing’, addressed in §E of the electronic supplementary material). In such a case, the majority of positive tests would be false positives, and the number of positive tests would increase with the overall number

3.2 $$N_{T}(t)=pI(t)+q[S(t)+E(t)+R(t)]$$

of tests made. Testing a large number of people without symptoms might, therefore, produce pretty arbitrary and misleading numbers, if appropriate statistical corrections are not done. As a consequence, the number of positive tests is not a good basis for policy measures. In particular, if the testing rate q is increased with the anticipated number of positive tests $N_{p}(t+\Delta t)$ at some future point in time t+Δt, this may lead to a ‘lock-in effect’. Then, for a long time, one may not find a way out of the vicious cycle of more predicted cases triggering more tests, which implies more measured and predicted cases, and so on.

It is much better to compare the number $N_{p}$ of positive tests given by (3.1) with the overall number of tests $N_{T}$, see (3.2). Accordingly, the measured proportion of Infectious people, also known as prevalence, is

3.3 s^(t)=Np(t)NT(t)=(1−β)pI+(1−β′)qE+αq(S+R)pI+q(S+E+R)

This equation might be seen as a naive estimate of the proportion of infectious individuals. Here, we have dropped the argument (t) on the right-hand side for simplicity. Let us now compare this measurement-based estimate with the actual (true) proportion of Infectious among living people, which is given by

3.4 $$s(t)=\frac{I(t)}{N^{\prime }(t)},$$

where

3.5 $$N^{\prime }(t)=N-D(t)=S(t)+E(t)+I(t)+R(t)$$

is the number of living people in the considered population. The relative error then becomes

3.6 $$\epsilon =\frac{|\hat{s}(t)-s(t)|}{|s(t)|}=\left|\frac{\left[(1-\beta )pI+(1-\beta^{\prime })qE+\alpha q(S+R)\right]}{\left[pI+q(N^{\prime }-I)\right]}\frac{N^{\prime }}{I}-1\right|.$$

If the tests measure the Exposed as if they were ‘still healthy’, it is reasonable to assume $(1-\beta^{\prime })=\alpha$.^1^ This simplifies the previous formula to

3.7 $$\epsilon =\frac{|\hat{s}-s|}{|s|}=\left|\frac{\left[(1-\beta )pI+\alpha q(N^{\prime }-I)\right]}{\left[pI+q(N^{\prime }-I)\right]}\frac{N^{\prime }}{I}-1\right|=\left|\frac{\left[(1-\beta )ps+\alpha q(1-s)\right]}{s[ps+q(1-s)]}-1\right|.$$

Let us investigate two limiting cases. First, if we assume that only people with symptoms are tested, then q=0 and

3.8 $$\epsilon =\frac{|\hat{s}-s|}{|s|}=\left|\frac{\left(1-\beta \right)}{s}-1\right|.$$

Accordingly, the smaller the actual proportion s(t) of Infectious, the bigger the relative error will be. Second, if the number $\left(N^{\prime }-I)=(S+E+R\right)$ of people without symptoms typically outweighs the Infectious by far, one may assume that $\alpha q(S+E+R)=\alpha q(N^{\prime }-I)\gg (1-\beta )pI$ and $q(N^{\prime }-I)\gg pI$. In this case, we get

3.9 $$\epsilon =\frac{|\hat{s}-s|}{|s|}\approx \left|\frac{\alpha }{s}-1\right|.$$

In both cases, we can see that the relative error increases with the inverse of the proportion s of Infectious. Accordingly, the relative error ϵ increases with the number of healthy people. This relative error might be huge. In fact, it is particularly big in cases of ‘mild’ epidemics characterized by s(t)≈0. Again, given the finite value of the false positive rate α, mass testing of people who feel healthy is not really advised, unless appropriate statistical corrections are done. It might lead to a large overestimation of the actual disease rate. That is, the state of the epidemic may be wrongly assessed.

However, there is a correction formula for the effect of false positives and negatives, and for biased samples with p≠q. Similar to the correction for testing errors and biases introduced in [20], to estimate the fraction of unhealthy people correctly, we need to find a function ${\hat{s}}_{c}$ that transforms the estimate $\hat{s}$ into s. Considering that the estimate $\hat{s}$ of s is given by (3.3) and that we have $s=I/N^{\prime }$ with $N^{\prime }=(S+E+I+R)$, under the previously made assumption $(1-\beta^{\prime })=\alpha$ we find

3.10 $$\hat{s}(s)=\frac{\left(1-\beta )ps+\alpha q(1-s\right)}{(p-q)s+q}.$$

From this, we can derive the corrected value ${\hat{s}}_{c}$ of $\hat{s}$ via the inverse function of $\hat{s}(s)$

3.11 s^c(s^):=s(s^)=(s^−α)q(1−β)p−αq−(p−q)s^.

This formula should only be used in the range $0<\hat{s}-\alpha <1-\alpha -\beta$. For non-biased samples (p=q), the formula simplifies to

3.12 $${\hat{s}}_{c}(\hat{s})=\frac{\hat{s}-\alpha }{1-\alpha -\beta }.$$

**Figure 1.**
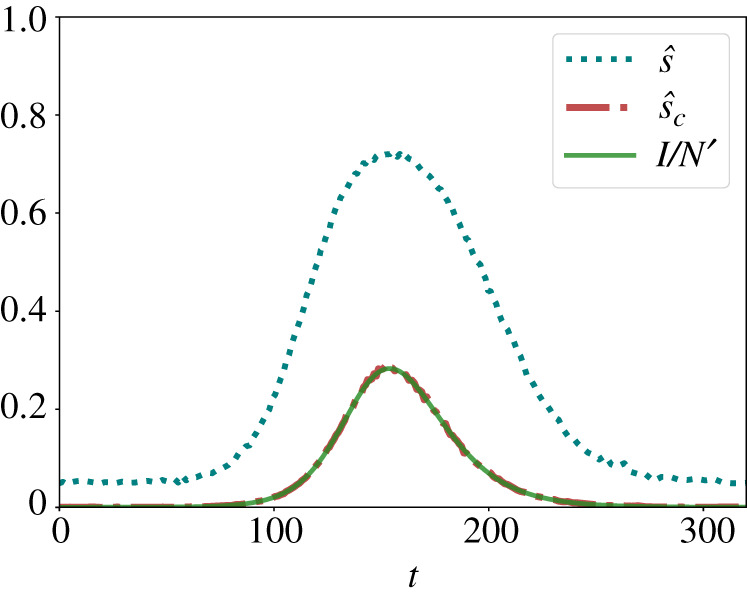
Estimation of the true proportion $I/N^{\prime }$ of Infectious (green solid line) based on the fraction (3.3) of positive tests (blue dotted line) and the correction formula (3.11) (red dashed-dotted line). The corrected estimate tends to be very close to the true value when the parameters α, β, p and q of the test method are known exactly. The monitoring parameters are: α=0.05, β=0.15, p=0.8, q=0.06. The curves displayed here are for SEIRD dynamics with N=100 000, a=1/5, b=3/14, c=1/28, d=1/28. (Online version in colour.)

As figure 1 shows, this correction can be very effective in fitting the true value $I/N^{\prime }$, if the parameters α, β, p and q are well known. However, we would like to point out that the above analysis and correction formula are based on a mean-value approximation. Let us, therefore, investigate the likely role of stochastic measurement errors. A common approach for this is the use of a binomial distribution. It describes the probability to have $n_{p}$ positive tests among $N_{T}$ tests, if the ‘success rate’ (of having a positive test result) is $\hat{s}$. Accordingly, the binomial distribution,

3.13 P(np|NT)=(NTnp)s^np(1−s^)NT−np,

where $N_{T}=pI+q(S+E+R)$ represents again the total number of tests made. For the binomial distribution, the expected number of confirmed cases is

3.14 $$\langle n_{p}\rangle =N_{T}\hat{s}=N_{p},$$

and the variance is

3.15 $$\text{Var}(n_{p})=N_{T}\hat{s}(1-\hat{s}).$$

As a consequence, the relative standard deviation is

3.16 $$\frac{\sqrt{\text{Var}(n_{p})}}{\left\langle n_{p}\right\rangle }=\frac{\sqrt{N_{T}\hat{s}(1-\hat{s})}}{N_{T}\hat{s}}=\frac{1}{\sqrt{N_{T}}}\sqrt{\frac{1-\hat{s}}{\hat{s}}}.$$

**Figure 2.**
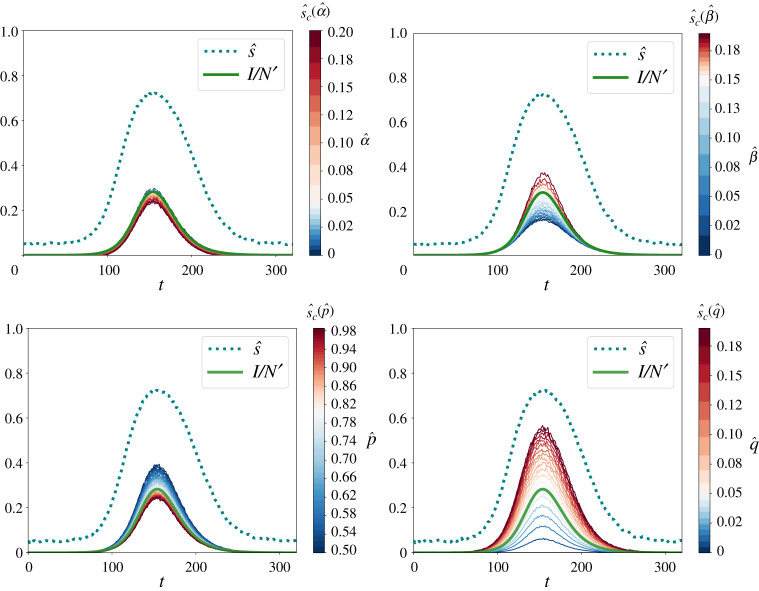
Estimation of the true proportion $I/N^{\prime }$ of Infectious (green solid line) based on the fraction (3.3) of positive tests (blue dotted line) and the correction formula (3.11) (other lines according to the colour scales on the right). In these figures, we assume that only three of the four parameters α=0.05, β=0.15, p=0.8 and q=0.06 are known exactly, while the estimate of the fourth one (represented with a hat on top) is uncertain and, therefore, varied. Specifically, in (3.11) we have replaced α by $\hat{\alpha }$ in the top left figure, β by $\hat{\beta }$ in the top right figure, p by $\hat{p}$ in the bottom left figure and q by $\hat{q}$ in the bottom right figure. All figures show results for a SEIRD dynamics with N=100 000, a=1/5, b=3/14, c=1/28, d=1/28. (Online version in colour.)

Here, more tests are better, as expected. While the relative standard deviation may be significant if $N_{T}$ is small (at the beginning of an epidemics) or if $\hat{s}\approx 0$, for a sufficiently large number of tests, the relative variance will be arguably small, likely around 1%. Therefore, the above mean value equations and correction formula (3.11) appear to be applicable in many cases, when the uncertainty in the measurement parameters α, β, p and q is low. In reality, however, the parameter values showing up in formula (3.11) are estimates $\hat{\alpha }$, $\hat{\beta }$, $\hat{p}$ and $\hat{q}$, which may deviate from the true parameter values α, β, p and q. Inserting these in formula (3.11) instead, one may find considerable under- or over-estimations of the true proportion of Infectious in a population (see figure 2 for the case p>q and figure 1 of §E in the electronic supplementary material for the ‘opposite’ case p<q, which corresponds to ‘mass testing’).

A further complication occurs, if the assumption $(1-\beta^{\prime })=\alpha$ does not hold (e.g. when the testing procedure recognizes Exposed as ‘already infectious’ people, even though they do not have symptoms yet). Then it is generally not possible to derive a correction formula such as (3.11). To derive such a formula, one would need to separately measure Infectious and Exposed, requiring two different, specific tests, but this is often not realistic to assume. Therefore, one would have to use additional assumptions or Bayesian inference.

## 4. Bayesian inference

Up to this point, we have assumed that the testing rates p and q are fractions determined by the health system. However, if these monitoring parameters reflect the probabilities of infectious and healthy individuals to be tested, and to be consequently observed as infected or not, we can consider them as random variables that vary from one test to another. Similarly, α and β are not fully known, but rather depend on factors such as the delay between the time an individual was exposed to a disease and being tested. It is worthwhile to note (although we do not consider this case here), that even the number of conducted tests is not exactly known, and likely varying with time.

To address these uncertainties, the monitoring parameters $\hat{\alpha },\hat{\beta },\hat{p},\hat{q}$ can be sampled from some independent distributions: $\hat{\alpha }∼f_{\hat{\alpha }},\hat{\beta }∼f_{\hat{\beta }},\hat{p}∼f_{\hat{p}},\hat{q}∼f_{\hat{q}}$ and used in modelling a likely number of Infectious I(t), given the number of positive tests $N_{p}$ at t. For example, $\hat{\alpha },\hat{\beta },\hat{p},\hat{q}$ may be Beta-distributed, which was the choice we studied here.

Considering that the number of Infectious and the number of positively tested people are also random variables in reality, Bayes’ rule applies. Here, our evidence is $N_{p}$ and our priors relate to knowledge about P(I)—the probability of the likely number of Infectious individuals. Bayes’ rule determines the probability that the hypothesis is true (here: the number of Infectious I is m), given a certain observation (here: the measured number n of positive tests)

4.1 $$P(I=m|N_{p}=n)=\frac{P(N_{p}=n|I=m)P(I=m)}{\sum_{l}P(N_{p}=n|I=l)P(I=l)}.$$

Here, the left-hand side of the equation is the ‘posterior’ probability that we aim to infer. Assuming that the binomial distribution in (3.13) for $N_{p}$ is well approximated by a normal distribution, that ${\hat{s}}_{i}=\hat{s}({\hat{p}}_{i},{\hat{q}}_{i},{\hat{\alpha }}_{i},{\hat{\beta }}_{i})$ for each sample i from the prior distributions $f_{\hat{\alpha }},f_{\hat{\beta }},f_{\hat{p}},f_{\hat{q}}$, and that all values of I are equally likely *a priori*, we can write the posterior probability distribution as

4.2 $$P(I=m|N_{p}=n)\;∼\frac{1}{M}\sum_{i=1}^{M}\left(\frac{1}{\sqrt{2\pi N_{T}{\hat{s}}_{i}(1-{\hat{s}}_{i})}}\exp\left(\frac{-{\left(n-N_{T}{\hat{s}}_{i}\right)}^{2}}{2N_{T}{\hat{s}}_{i}(1-{\hat{s}}_{i})}\right)\right).$$

In practice, this formulation allows us to employ Monte Carlo sampling to infer the desired probability distribution efficiently. Overall, we consider the Bayesian posterior estimate to be

4.3 I^=⟨P(I=m|Np=n)⟩=∑m=0NmP(I=m|Np=n),

where ⟨.⟩ denotes the expectation over the values of $P(I=m|N_{p}=n)$. The full derivation of (4.3) is provided in §C of the electronic supplementary material.

Last but not least, network effects may also produce significant deviations from the mean-value approximations above (which implicitly assumed homogeneous mixing and a low variance of measurement errors). On the other hand, good knowledge of a contact network may be of use when inferring $\hat{I}$: sparse networks slow the disease spread, dense or small-world networks may speed it up. To assess the potential benefits of network priors as well as the extent to which the network can introduce a new source of error, in the following section, we will use a more sophisticated simulation approach for the SEIR/SEIRD model that considers stochastic as well as network effects.

To include *network priors* in the Bayesian framework, we will consider samples of epidemic trajectories from $N_{G}$ randomly generated networks with given network characteristics. For each of the networks, we simulate $N_{e}$ epidemic trajectories, each with a randomly chosen initial infectious node, giving a prior distribution for P(I=m). The prior distribution $P(I_{t}=m)$ is then estimated as the probability density function of trajectories in the time window [t−δ,t]. We can incorporate this network prior in (4.3) such that $P(I=m|N_{p}=n)∼P(I=m|N_{p}=n)P(I=m)$ for each sample i.

## 5. Stochastic simulation of epidemic spreading in social networks

In order to study stochastic SEIRD dynamics, we will consider epidemic spreading on a contact network G=(V,E), defined by a set of nodes V (corresponding to N individuals) and a set of edges E (representing their contacts, M in total). Here, we are interested in the SEIRD process, determined by the parameters (a,b,c,d), where a Susceptible individual becomes Exposed with rate b by having a contact with an Infectious neighbour, Exposed transition from the Exposed state to Infectious state with rate a, and Infectious Recovered or Died with an overall rate c+d. These processes have exponential inter-event time distributions $\psi (\tau )=be^{-b\tau }$ (spreading) and $\phi (\tau )=(c+d)e^{-(c+d)\tau }$ (recovery or death), $\xi (\tau )=ae^{-a\tau }$ (transition from Exposed to Infectious).

To simulate a set of $N_{e}$ possible stochastic SEIRD epidemic spreading outcomes on a given contact network G, we use the SP-KMC shortest-path Kinetic Monte Carlo method described in [27,28] for the SIR model, and expand it to the SEIRD model. For both SIR and SEIRD models, the SP-KMC method works in two steps: (i) creating weighted networks $\left\{G_{N_{e}}^{\mathrm{SEIRD}}\right\}$ and (ii) extracting a set of k possible dynamic realizations from $\left\{G_{N_{e}}^{\mathrm{SEIRD}}\right\}$, where the superscript indicates the type of epidemic model considered.

We then proceed with the first step by building a *time-respecting weighted network* instance $G_{N_{e}}^{\mathrm{SEIRD}}$ created by taking the input network G and assigning weights to the edges of the network instance such that

5.1 $$\rho_{ij}=\left\{ \begin{array}{ll} -\ln(x)/b-\ln(z)/a & \text{if }-\ln(x)/b\leq -\ln(y)/(c+d)\\ \infty & \text{if }-\ln(x)/b>-\ln(y)/(c+d) \end{array}\right.$$

Here, x,y,z are uniform random numbers from the domain [0,1]. However, the SP-KMC method can also work with non-exponential inter-event distributions [27,28]. Each weighted network $G_{N_{e}}^{\mathrm{SEIRD}}$ is linked to a possible outcome of the epidemic spreading process, starting from a randomly selected ‘source’ node. Therefore, to extract an epidemic trajectory represented by $G_{N_{e}}^{\mathrm{SEIRD}}$, one needs to find the shortest paths from the source node to other nodes in this network [27,28]. We define the distance as the shortest path on the weighted network

5.2 $$d_{G_{N_{e}}^{\text{SEIRD}}}(v_{i},v_{j})=\min_{\chi_{ij}}\sum_{(k,l)\in \chi_{ij}}\rho_{kl},$$

where $\chi_{ij}$ is the set of all possible paths from node $v_{i}$ to node $v_{j}$ on the network $G_{N_{e}}^{\mathrm{SEIRD}}$, and $\rho_{kl}$ denotes the weights defined in (5.1). Since the length of the shortest path from the source to some other node represents the first infection time in epidemic realization, the temporal evolution of the number of infectious is extracted from the statistics of the shortest paths in $G_{N_{e}}^{\mathrm{SEIRD}}$ (see §D of the electronic supplementary material for more details).

### (a) Network models

We studied random networks generated using two network models: (i) the Barabási–Albert model and (ii) the Erdös–Rényi model. The Erdös–Rényi (ER) network model [29] is probably the simplest random network model. In this model, one assumes no prior knowledge about the network, nodes and edges. To build a representative network of this model, one follows a simple rule: consider each pair of nodes, and with some probability π build an edge between them: each pair of nodes is connected with a probability π to give a simple graph. ER networks are ‘structure-less’, being rather dissimilar to most real-world networks [30]. Given π and the number of nodes, N, it is useful to characterize this network by the average degree of nodes, ⟨k⟩, equal to the average number of links adjacent to a node. For a large ER network, ⟨k⟩=Nπ and node degrees are Poisson-distributed.

Another network type we consider is called ‘scale-free’; here, the degree distribution follows a power law [31]. The term ‘scale-free’ means that there is no typical number of edges. As a result, we have many nodes with very small degree (close to one), and very few ‘winner’—nodes with very large degrees (we say that the degree heterogeneity is large) [30].

The most famous mechanism to generate scale-free networks is preferential attachment. In preferential attachment, the more connected a node is, the quicker it acquires more new connections. The Barabási–Albert (BA) [32] is one model that generates random scale-free networks using a preferential attachment mechanism. A BA network is generated iteratively, beginning with an initial connected network of μ nodes. Each new node is connected to μ existing nodes with a probability π that is proportional to the degrees of existing nodes, and this probability is defined as $\pi \propto k^{\gamma }+c$. Here, c can be interpreted as a number of edges out of μ that are attached via random attachment at each time step. If γ=1, the resulting network is scale-free and $P(k)\propto k^{-3}$ [33].

### (b) Results with mean-field correction

**Figure 3.**
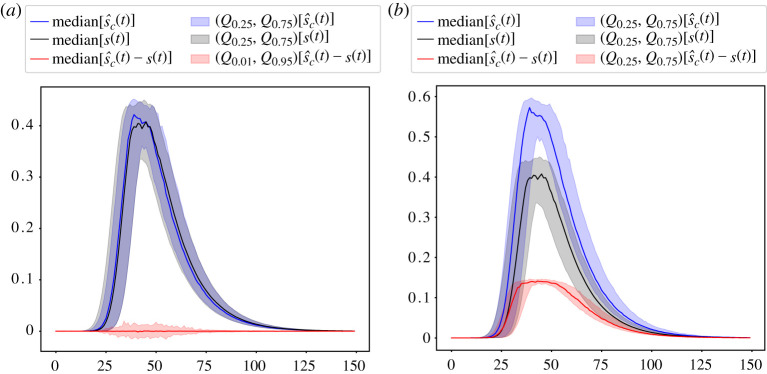
Ensemble of stochastic trajectories (SP-KMC sampling) on an ensemble of $10^{3}$ Barabási-Albert networks with N=100 000, μ=3 and γ=1. (*a*) Accurate epidemic reconstruction assuming that the parameters α=0.05, β=0.15, p=0.8, q=0.06 of the test method are exactly known. (*b*) Inaccurate reconstruction for somewhat incorrect estimates $\hat{p}=0.75$ and $\hat{q}=0.1$, while the other parameters are assumed to be the same. The assumed parameters of the SEIRD dynamics are: a=1/5, b=3/14, c=1/28, d=1/28. $Q_{0.01}$, $Q_{0.25}$, $Q_{0.75}$ and $Q_{0.99}$ represent 1, 25, 75 and 99 per cent quantiles. The median values and error quantile bands are based on $10^{3}$ simulations. (Online version in colour.)

Figure 3 shows on the left that the mean-field correction (3.11) works well if all measurement parameters α, β, p and q are known. However, for somewhat incorrect parameter estimates, we observe considerable deviations from the mean-field correction. This establishes the need for Bayesian inference.

### (c) Results for Bayes correction with measurement priors

**Figure 4.**
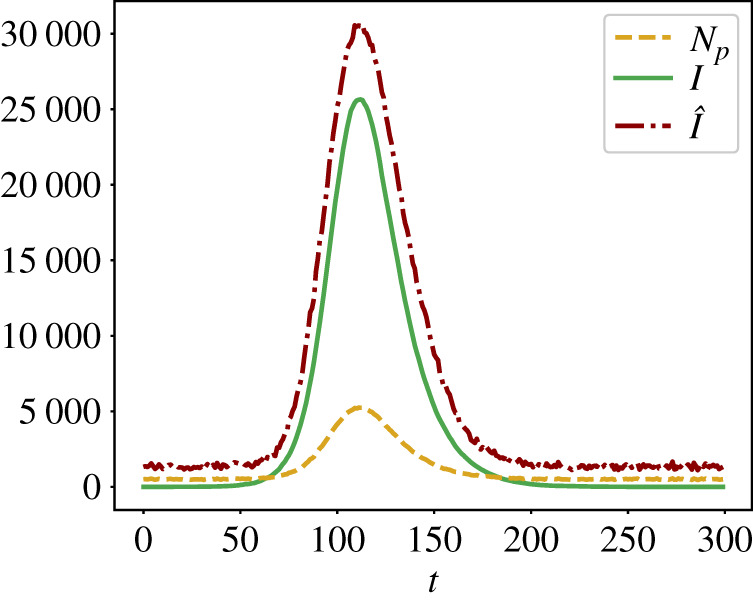
$\hat{I}(t)$ computed from (4.3), assuming uncertainty in monitoring parameters: $\hat{\alpha }∼\mathrm{Beta}(10,90)$, $\hat{\beta }∼\mathrm{Beta}(5,95)$, $\hat{p}∼\mathrm{Beta}(4,16)$, $\hat{q}∼\mathrm{Beta}(5,95)$. We used the distributions whose mean is ‘guessed’ correctly, i.e.$\langle \hat{\alpha }\rangle =\alpha$. The true number I(t) of Infectious was obtained by simulating SEIRD dynamics with parameters a=1/5, b=3/14, c=1/28, d=1/28 on an ER network with $N=10^{5}$ and average degree 1.5. (Online version in colour.)

Figure 4 shows that, using the Bayesian inference (4.3), one can improve the mean-field correction (3.11), when monitoring parameters are not fully known. Here, we consider a case when α,β,p,q are all assumed to be unknowns, sampled from independent Beta distributions, see caption of figure 4. Furthermore, in this case, we assume that the Beta distributions have correct mean values, i.e. $\alpha =\langle \hat{\alpha }\rangle$ and similarly for β,p and q. Despite some minor over-estimation, the Bayesian correction can work well in the case when the priors are appropriate.

### (d) Results for Bayes correction with network priors

**Figure 5.**
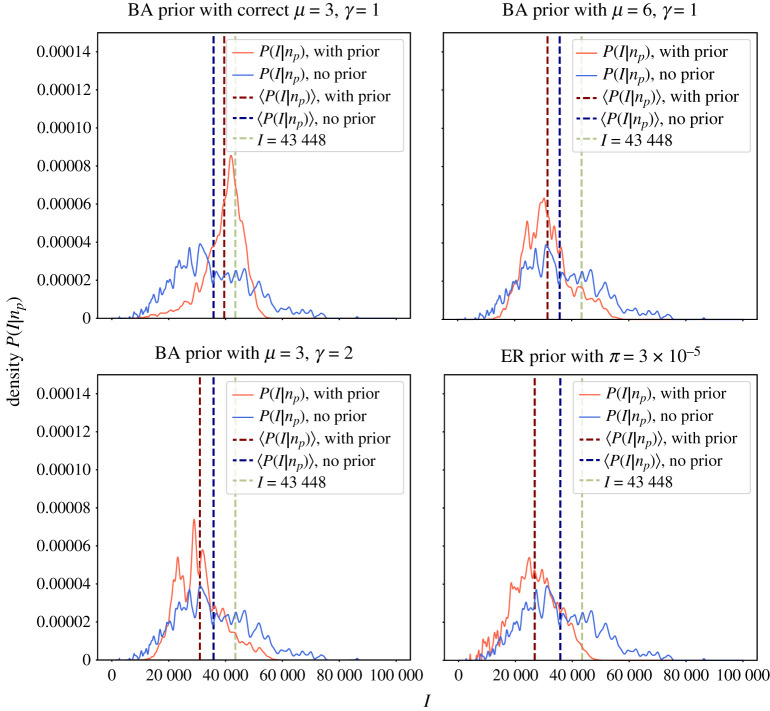
Posterior distribution $P(I|n_{p})$ when a network topology prior is used (in red) and when it is not (in blue). Monitoring parameters were also considered as priors, with $\hat{p}∼\mathrm{Beta}(4,16)$, $\hat{q}∼\mathrm{Beta}(5,95)$, $\hat{\alpha }∼\mathrm{Beta}(5,95)$, $\hat{\beta }∼\mathrm{Beta}(10,90)$. For the ground truth parameters, we selected α=0.05, β=0.1, p=0.2, q=0.05. The ground truth number of Infectious I=43 448 was obtained from an epidemic trajectory generated on a network sampled from an ensemble of Barabási–Albert (BA) networks with μ=3, γ=1, giving a scale-free network with an average degree ⟨k⟩=3. In scenario ‘BA prior with correct μ=3, γ=1’, we used a prior which assumes the correct network ensemble—Barabási-Albert networks with N=100 000, μ=3 and γ=1. In ‘BA prior with μ=6, γ=1’, we used a network ensemble prior of BA networks with μ=6 and γ=1, which over-estimates the average node degree ⟨k⟩. In ‘BA prior with μ=3, γ=2’, we used a prior for BA networks with μ=3 and γ=2, and in ‘ER prior with $\pi =3\times 10^{-5}$’ we used a prior for Erdös–Rényi networks with $\pi =3\times 10^{-5}$, giving networks with ⟨k⟩=3, but the degree distribution P(k) is Poisson rather than a power law. In all figures, the green line shows the ‘ground truth’, against which the biased testing is performed. We observe that knowledge about the degree distribution of contacts helps to estimate the true ground truth. To estimate network priors within t∈[60,65], we have used 1000 networks from the ensemble. In all cases, M=1000 Monte Carlo samples were used to estimate (4.3). (Online version in colour.)

In figure 5, we show that using stochastic trajectories generated with networks from a correct ensemble of networks, one can improve the estimation of $\hat{I}=\hat{s}N^{\prime }$. However, when using a prior with wrong assumptions about the degree distribution of contacts, we see no improvement in the posterior estimate of the ground truth, compared to the uninformative prior. Overall, we find that both the degree distribution and the network density have effects on the quality of the Bayesian inference. Therefore, we would like to emphasize the importance of choosing correct priors for a reliable estimation of the ground truth.

For the example shown in figure 5, we considered networks with N=100 000 nodes. The ground truth networks were generated using a BA model with μ=3, γ=1, yielding scale-free networks. To study the implications of using somewhat incorrect network priors, we used networks generated with either a BA model and wrong average degree (here, we used μ=6) and wrong γ (here, we used γ=2). We also considered the case when the network prior is assumed to be an ER model generated network with $\pi =3\times 10^{-5}$, yielding an average degree of 3 in the network.

## 6. Summary, conclusion, discussion and outlook

In this paper, we addressed some challenges regarding epidemic monitoring and modelling, particularly regarding (i) the modelling itself and (ii) the applicability of models to the real world. Regarding the first point, it is important to choose the right epidemiological model and to consider limitations of the mean value-based correction approach. Stochastic and network effects—in combination with the underlying nonlinear spreading dynamics—are relevant as well. Regarding the second point, one needs to consider errors of tests and estimation procedures underlying epidemic monitoring. This concerns false-positive and false-negative rates as well as biases in the measurement sample (p≠q). Our goal was to analyse a good approximation of reality (where we have represented the latter by an assumed ground truth based on SEIRD dynamics). Overall, we showed that data-driven approaches relying only on measured numbers of Infectious can be misleading, if reliable measurement corrections are not available or not applied.

Note that the presented Bayesian inference also makes hidden modelling assumptions such as (i) testing errors are distributed binomially or normally, (ii) parameters are time-independent, (iii) all relevant parameters are being considered in the Bayesian analysis. Under these assumptions, we have found several limitations of different statistical methods for inferring the hidden state of epidemics.

Monitoring real-world epidemics is even more difficult, because one does not know the ground truth, and the goal of all types of modelling is to infer it. Although a simple mean-field correction can work pretty well if the measurement parameters are known (figure 1), substantial deviations may occur when there is some uncertainty about them (figure 2), which is usually the case. Then, one might resort to a Bayesian correction for improvements [20–24], but this will not completely remove all issues of measurement errors and uncertainties either.

For all the above reasons, forecasting the epidemic dynamics is a problem that has fundamental limitations even in the age of Big Data and Artificial Intelligence. Given the intrinsic limitations of tests, these problems are expected to stay around. Measurement problems using dynamical models [14,34] may be further amplified if the test method or the social behaviour of people are dynamically changed in response to the epidemic dynamics. Hence, data protection regulations are not the main problem for monitoring, forecasting and controlling epidemics. The limitations of a data-driven approach are a lot more fundamental in nature.

## Footnotes

1 Because Exposed tested positive would then be considered ‘false positives’—they would be valued ‘true positive’, once they have transitioned to the state of ‘Infectious’.

## Data accessibility

This paper is not presenting any empirical data. Additional information is available in the electronic supplementary material.
